# Changes to Extender, Cryoprotective Medium, and *In Vitro* Fertilization Improve Zebrafish Sperm Cryopreservation

**DOI:** 10.1089/zeb.2017.1521

**Published:** 2018-06-01

**Authors:** Jennifer L. Matthews, Joy M. Murphy, Carrie Carmichael, Huiping Yang, Terrence Tiersch, Monte Westerfield, Zoltan M. Varga

**Affiliations:** ^1^ Zebrafish International Resource Center, University of Oregon, Eugene, Oregon.; ^2^ Aquatic Germplasm and Genetic Resources Center, School of Renewable Natural Resources, Louisiana State University Agricultural Center, Baton Rouge, Louisiana.

**Keywords:** aquatic, biomedical, gene banking, genetic repository, model organism, resource center

## Abstract

Sperm cryopreservation is a highly efficient method for preserving genetic resources. It extends the reproductive period of males and significantly reduces costs normally associated with maintenance of live animal colonies. However, previous zebrafish (*Danio rerio*) cryopreservation methods have produced variable outcomes and low post-thaw fertilization rates. To improve post-thaw fertilization rates after cryopreservation, we developed a new extender and cryoprotective medium (CPM), introduced quality assessment (QA), determined the optimal cooling rate, and improved the post-thaw *in vitro* fertilization process. We found that the hypertonic extender E400 preserved motility of sperm held on ice for at least 6 h. We implemented QA by measuring sperm cell densities with a NanoDrop spectrophotometer and sperm motility with computer-assisted sperm analysis (CASA). We developed a CPM, RMMB, which contains raffinose, skim milk, methanol, and bicine buffer. Post-thaw motility indicated that the optimal cooling rate in two types of cryogenic vials was between 10 and 15°C/min. Test thaws from this method produced average motility of 20% ± 13% and an average post-thaw fertilization rate of 68% ± 16%.

## Introduction

Germplasm cryopreservation is crucial for the storage and availability of genetic resources for biomedical research. In recent years, the zebrafish (*Danio rerio*) research community has generated novel mutations and transgenic lines at an unprecedented rate, producing an enormous resource for studying gene function. Several large-scale mutagenesis programs have produced tens of thousands of novel mutations in the zebrafish genome by exploiting advances in sequencing technologies and the availability of a genome map and sequence.^1–4^ With the recent advent of efficient genome editing technologies,^5,6^ even more genetic modifications of the zebrafish genome are being produced. Consequently, the genetic inventory at the Zebrafish International Resource Center (ZIRC), a central repository of genetic zebrafish lines, has increased significantly in number^7^ and genetic complexity.^8^ Resource centers and research laboratories alike are faced with the challenge of maintaining a vast and increasing number of fish lines. Cryopreservation of sperm is a proven method for long-term storage and reducing maintenance costs of genetic materials.

The first cryopreservation protocol for zebrafish sperm was published in 1982^9^ and was used throughout the zebrafish community.^10^ It was modified for high-throughput mutation screens^11,12^ and adopted by ZIRC^13^ and other laboratories. Through systematic evaluation of cryoprotectants, two additional protocols were developed that utilized modified cryoprotective mediums (CPMs) and extenders.^14,15^ All of these protocols produced comparable, although variable, post-thaw motility and fertilization rates.

Post-thaw outcomes have been shown to be affected by factors such as damage to cells due to solution toxicity, osmotic stress, and extra- and intracellular ice formation during the freezing and thawing processes.^16^ Previous zebrafish cryopreservation research^11,12,14^ was primarily focused on post-thaw outcomes, and the cryobiological principles of the resulting variability remained poorly understood. To ensure recovery, research facilities and resource centers required large numbers of cryopreserved samples per line, limiting the overall efficiency of cryopreservation.

One source of variation in fertilization rates could be the premature osmotic activation of sperm cells during collection. The sperm of most freshwater fishes are immotile in testes and seminal plasma and become activated when released into a hypotonic aqueous environment during spawning.^15,17–20^ Motility can be started and stopped by repeated adjustment of diluent buffer (extender) osmolality.^20–22^ For zebrafish, sperm motility can be initiated in hypotonic solutions below 270–288 mmol/kg.^15,23^ Other studies and our own observations indicated that sperm can be contaminated with urine during collection by stripping or abdominal massage. This can lead to premature activation and variable reduction in fertilization rates.^24–26^ For an improved protocol, it was necessary to optimize and control the osmolality of solutions^27^ and to critically evaluate all the steps of the cryopreservation and *in vitro* fertilization (IVF) procedures.

We report a new protocol for cryopreservation of zebrafish sperm for resource center and laboratory use that refines and optimizes several elements of the cryopreservation process. It features quality assessment (QA)^27^ for cell density and motility, improved extender composition, a new CPM, effective freezing and thawing procedures for two types of cryogenic vials, and improved IVF conditions for thawed samples. This method is more efficient than previous methods and produces consistently high post-thaw fertilization rates while offering more flexibility. It can be performed alone or in teams. Sperm can be collected by stripping or testis dissection from single males or pooled from multiple males, and samples can be processed individually or in large batches. The protocol can be adapted to suit individual research facility needs.

## Materials and Methods

### Zebrafish

AB wild-type zebrafish from the ZIRC breeding facility were maintained in standard conditions as previously described at 28.5°C on a recirculating water system.^10,28–30^ Seven- to 14-month-old fish were fed as previously described and received an additional dry-food feeding at midday.^29^ Research and animal care were approved by the University of Oregon Institutional Animal Care and Use Committee, Protocol #15-05.

### E400 extender composition

The E400 sperm extender was developed to prevent premature sperm activation by a hypotonic environment and for holding and diluting sperm before cryopreservation. We tested several previously published extender components^14,15,31,32^ and refined the composition of our extender in an iterative step-by-step process. The beneficial characteristics of glucose and potassium had been suggested in previous studies; therefore, we tested only for the presence and absence of these components. In addition, we tested several buffering agents for sperm toxicity and holding times on ice before cryopreservation (TES, TES-Tris, HEPES, Tricine, Bicine, and HEPES). We chose HEPES buffer because of its low cell toxicity and reported buffering stability at ultralow temperatures.^33^ Potassium concentration was raised to achieve an osmolality well above our established activation threshold of around 300 mmol/kg.^15^

Our current E400 extender is composed of 130 mM KCL, 50 mM NaCl, 2 mM CaCl_2_, 1 mM MgSO_4_, 10 mM D-(+)-Glucose, and 30 mM HEPES-KOH (pH 7.9), resulting in an osmolality of ∼400 mmol/kg. E400 was used to normalize the volume of collected sperm in the microcapillary, or sperm was added directly into a predetermined volume of E400 at the time of collection. A microcentrifuge tube containing E400 was prepared and placed on ice before sperm collection. The volume of E400 for suspending sperm was based on the number of males to be stripped (5–10 μL/male) or the number of samples to be generated (5 μL/sample).

### Sperm collection by stripping

Males were anesthetized by immersion in 168 mg/L tricaine methanesulfonate solution (MS-222; Western Chemical, Inc.) and briefly rinsed in fish water or in a phosphate-buffered saline (PBS: pH 7.4 powder packets, Sigma #P3813, dissolved in 870 mL four-stage deionized, reagent grade water [dH_2_0]; final osmolality 305–315 mmol/kg) to establish isotonic conditions. The fish were gently dried by rolling on a paper towel and placed in dorsal recumbency (belly up) in the slit of a dampened foam holder. The urogenital opening was further dried with a cotton-tipped swab or Kimwipe just before sperm collection. Stripped sperm was collected into a 10-μL calibrated glass microcapillary (# 2-000-010; Drummond) using gentle, bilateral abdominal pressure with Millipore filter forceps (# XX6200006P; Millipore). Fish were transferred to fresh fish water for recovery immediately after stripping.

### Motility testing of freshly stripped sperm

To test for premature sperm activation, sperm was collected into a 10-μL microcapillary without extender, immediately expelled onto a hemocytometer counting chamber, covered with a glass coverslip, and observed with a compound microscope at 100 × magnification (*n* = 10 males).

### Osmolality determination of cell-free seminal plasma and blood plasma

Sperm was collected and pooled from 40 males by stripping, and cells were pelleted by centrifugation at 20,800 *g* (rcf) at 4°C for 5 min. The supernatant was removed and centrifuged again at 20,800 *g* at 4°C for 5 min. Ten microliters of the supernatant (seminal plasma) was analyzed with a vapor pressure osmometer (VAPRO Model 5520; Wescor, Inc.). To replicate the test, cell-free supernatant was obtained and analyzed with sperm stripped and pooled from 50 additional males. Blood was collected from 16 hypothermal-shock-euthanized zebrafish into two heparinized capillary tubes by severing the caudal peduncle. Blood plasma was separated from cells by centrifugation in a microhematocrit centrifuge at full speed (10,000 rpm) for 10 min. Ten microliters of supernatant (blood plasma) was analyzed with the vapor pressure osmometer.

### Sperm cell density measurement

Sperm concentration was measured with a Thermo Scientific NanoDrop 2000 spectrophotometer using the *Cell Culture* program provided by the manufacturer. Each sample was diluted in E400 by 1:5 or 1:10. 1.5 μL of the dilution was analyzed thrice, and results were averaged. Cell concentration was calculated from the averaged absorbance at 400 nm (A_OD400_). The NanoDrop 2000 was calibrated with a hemocytometer-generated standard curve and equation.^34^ The best fit (*R*^2^ = 0.982) between data and fitted curve resulted from a second-order polynomial equation:

\begin{align*}
 { \frac { { \rm { cells } } }  { { \rm { mL } } } } = { \rm { \; } } 5 \cdot { 10^8 } \cdot { ( { { \bf { A } } _ { { \rm { OD } } 400 } } ) ^2 } + 7 \cdot { 10^7 } \cdot ( { { \bf { A } } _ { { \rm { OD } } 400 } } ) + 2 \cdot { 10^7 } \tag { 1 } 
\end{align*}

The equation was used in an Excel calculator to determine cell density based on absorption: (http://zebrafish.org/zirc/documents/protocols/xls/cryopreservation/zirc_nanodrop_sperm_density_calc.xls).

### Cryoprotectant RMMB and sperm cryopreservation in dry ice

We tested several previously published cell-penetrating^14,15^ (Methanol, N,N-Dimethylformamide (DMF), Dimethyl sulfoxide (DMSO), N,N-Dimethylacetamide (DMA), and 1-Thioglycerol) and nonpenetrating (Sucrose, Trehalose, and Raffinose) cryoprotective agents (CPAs) with our new extender E400 for their sperm toxicity and holding times on ice, in an iterative step-by-step process. We excluded DMF because of its cytotoxic characteristics and excluded DMSO, DMA, and 1-Thioglycerol because we did not observe any significant improvement in post-thaw sperm motility. We continued to use methanol as a CPA because we confirmed its low cytotoxicity as shown in previous studies.^11,15^ However, we decreased the final methanol concentration further than in previously published protocols.

The RMMB CPM consists of 20% w/v D-[+]-Raffinose pentahydrate (R7630; Sigma), 2.5% w/v Skim Milk (#232100; Difco), 6.67% v/v Methanol (absolute, acetone-free, certified ACS reagent grade, Fisher Scientific A412-500), and 30 mM Bicine buffer (B3876; Sigma), pH adjusted to 8.0 with NaOH. Before cryopreservation, cell density was adjusted with E400 to 4–8 × 10^8^ cells/mL, and three parts of RMMB were added to one part sperm/E400 suspension. Sperm and RMMB were mixed by gentle pipetting and 20 μL aliquoted into each cryogenic vial (0.5-mL Matrix screw top storage tubes, Thermo Scientific #3745-BR or 2-mL Corning vials #430488). Samples were frozen in a controlled-rate freezer (CRF) or in dry ice. For freezing in dry ice, the sample vial was placed on top of an empty spacer vial in a 15-mL conical centrifuge tube (Falcon 352096; Fig. 3B). The conical tube was capped, driven into powdered dry ice (produced as previously described^13^), and held for 20–60 min. Samples were then transferred to liquid nitrogen for storage.

### Sperm thawing and sperm solution SS300

Sperm samples were warmed in a water bath at 38°C for 10–15 s. Just before the sample completely thawed, 150 μL of room temperature (25°C–28°C) sperm solution SS300 (140 mM NaCl, 5 mM KCl, 1 mM CaCl_2_, 1 mM MgSO_4_, 10 mM D-[+]-Glucose, and 20 mM Tris-Cl, pH 8.0, 300 mmol/kg) was added to the sample vial.

### Sperm motility analysis

Sperm motility was analyzed using a Hamilton Thorne CEROS II computer-assisted sperm analysis (CASA) System and a Makler^®^ counting chamber (10 μm fixed depth, plain coverslip; Sefi Medical Instruments Ltd.). For CASA, all sperm was diluted to match IVF solution conditions. Frozen sperm samples were thawed as described above. Fresh sperm was diluted 1:33 μL with SS300 containing 10% RMMB. For analysis, 5 μL of dH_2_O was loaded onto the surface of a Makler slide; 4.25 μL of the diluted sperm sample was added, mixed, and immediately covered with the coverslip. Motility was recorded using a digital camera and negative phase contrast (dark field) at 100 × magnification. For each sample, triplicate analyses were made, and for each analysis four videos (30 frames each) were captured at 60 Hz and analyzed with the Animal Motility Software (Version 1.6.3; Hamilton Thorne, Beverly, MA).

### Stability of pooled sperm in E400 sperm extender

Sperm from 18 males was collected by stripping and pooled for this experiment. The sperm volume collected from each male (ranging from 0.5 to 2 μL) was adjusted to 5 μL with E400 (normalized) before pooling. After pooling, the sperm concentration (1.7 × 10^9^ cells/mL) was determined by optic density measurements with the NanoDrop. The pooled sperm was split, and one half remained without further dilution at 1.7 × 10^9^ cells/mL on ice. The other half was diluted further to 5.0 × 10^8^ cells/mL with additional E400 (a 3.4 × additional dilution), aliquoted into six microcentrifuge tubes (21 μL each), and held on ice. One 5.0 × 10^8^ cells/mL aliquot was processed every hour for 6 h. Processing consisted of using 1 μL of sperm for prefreeze CASA and cryopreserving three samples from the remaining 20 μL, as described above. The undiluted control pool at 1.7 × 10^9^ cells/mL was processed in parallel. Every hour before freezing, a portion of the control was removed and diluted to 5.0 × 10^8^ cells/mL with E400. From this dilution, 1 μL was used for prefreeze CASA, and three samples were cryopreserved as above.

### RMMB CPM equilibration time

Stripped sperm was collected from 15 males into 100 μL of E400. Sperm cell concentration was adjusted to 8 × 10^8^ cells/mL with E400. The sperm suspension was divided into eight tubes (20 μL each) and held on ice until RMMB was added. Sixty microliters of RMMB was mixed with each of the 20-μL sperm aliquots and held at 25°C for a predetermined equilibration time, ranging from 0.5 to 60 min. At the end of each equilibration time point, 20-μL samples were aliquoted into three 2-mL cryogenic vials (#430488; Corning) and frozen in dry ice as previously described. As a control treatment, sperm was held at 25°C in E400 without RMMB. After 60 min, RMMB was added, and after an equilibration time of 0.5 min, three samples were frozen. The experiment was repeated thrice, and results were averaged.

### Determination of optimal cooling rate with a CRF and dry ice

Stripped sperm was pooled from 32 males into 150 μL of E400, diluted to 8.0 × 10^8^ cells/mL with E400, and held on ice as nine 26-μL aliquots. Immediately before RMMB addition, 1 μL of sperm was removed for CASA prefreeze motility analysis. Seventy-five microliters of 25°C RMMB was added to the remaining 25 μL of sperm, mixed, and aliquoted into four 20 μL samples (0.5-mL Matrix tubes or 2-mL Corning vials). Samples were frozen in a cryogenic CRF (CryoMed Model 7456; Thermo Electron Corporation) or in dry ice. From each batch of samples frozen, one sample was used for temperature measurements using a thermocouple (Type T, 0.010″ Teflon insulated, copper-constantan; Omega), and the CRF software recorded temperature every 6 s throughout the cooling process. The CRF was programmed to ramp down the chamber temperature from 25°C to −78°C at predetermined cooling rates ranging from 5 to 35°C/min. At the end of the cooling program, the samples were transferred into liquid nitrogen. For dry-ice freezing, samples were frozen in cryogenic vials (0.5-mL Matrix or 2-mL Corning) in a 15-mL conical centrifuge tube as previously described. After the sample temperature reached −78°C (∼15 min), samples were removed from the dry ice and transferred into liquid nitrogen. The motile cell survival rate was calculated based on prefreeze and post-thaw motile cell counts and represents the percentage of prefreeze motile cells remaining post-thaw (Table 1).

**Table 1.** Controlled-Rate Cooling Rates, Post-thaw Cell Motility, and Post-thaw Motile Cell Survival Are Comparable to Freezing in Dry Ice with Two Types of Cryogenic Vials

*Prefreeze*					*Post-thaw*		
*CRF ramp rate setting (°C/min)*	*Vial type*	*Motility (%)*	*Motile cell count (M/sample)*	*Measured cooling rate*	*Motility (%)*	*Motile cell count (M/sample)*	*Motile cell survival (%)*
−5	0.5-mL Matrix	97.8 ± 0.4	30.7 ± 9.2	−5.6	3.3 ± 3.6	0.2 ± .02	0.7
−10	0.5-mL Matrix	98.3 ± 0.6	38.7 ± 5.9	−10.4	74.0 ± 10.5	11.0 ± 3.6	28.4
−15	0.5-mL Matrix	98.0 ± 0.5	36.8 ± 6.5	−14.8	75.5 ± 5.2	11.5 ± 2.9	31.3
−20	0.5-mL Matrix	97.3 ± 0.6	33.3 ± 5.3	−19.8	60.5 ± 5.5	6.3 ± 1.0	18.9
−25	0.5-mL Matrix	97.4 ± 1.2	29.9 ± 1.6	−25.6	56.4 ± 8.7	5.9 ± 2.1	19.7
−30	0.5-mL Matrix	96.8 ± 1.2	31.6 ± 6.6	−28.7	48.6 ± 5.5	4.1 ± 0.7	13.0
−35	0.5-mL Matrix	97.4 ± 1.0	34.5 ± 5.3	−31.5	48.5 ± 4.9	4.6 ± 0.8	13.3
Dry ice	0.5-mL Matrix	97.1 ± 0.2	31.4 ± 1.1	−14.1	81.7 ± 2.5	14.0 ± 2.8	44.6
	2-mL Corning	96.5 ± 1.1	26.1 ± 8.7	−16.1	74.2 ± 3.5	10.1 ± 2.0	38.7

Cooling rates, average percent motility, and motile cell counts (M/sample = million cells/sample) of prefreeze and post-thaw sperm frozen in a CRF or in dry ice. Percent cell survival is the percent of prefreeze motile cells remaining post-thaw.

CRF, controlled-rate freezer.

### Mixing of eggs and sperm during fertilization

A clutch of eggs was divided into two Petri dishes, and each half was fertilized with thawed sperm samples from the same male (*n* = 20). After thawing in a 33°C water bath, 70 μL of HBSS300 (137 mM NaCl, 5.4 mM KCl, 1.3 mM CaCl_2_, 1.0 mM MgSO_4_, 0.25 mM Na_2_HPO_4_, 0.44 mM KH_2_PO_4_, 4.2 mM NaHCO_3_, and 5.55 mM Glucose, pH 7.2, ∼300 mmol/kg) was added to each cryovial, and each mixture was transferred to one of the two egg dishes. In each dish, sperm was activated with 750 μL dH_2_O. Immediately after activation, one dish was swirled for 10 s to mix sperm and eggs (Draper–Moens Cryo/IVF, Table 2). The other dish was left undisturbed for 2 min. The average fertilization rate of each test group was determined based on development at 3 hours post fertilization (hpf).

**Table 2.** Summary of Zebrafish International Resource Center Sperm Cryopreservation, Thawing, and *In Vitro* Fertilization Protocol Changes from 2004 to 2014

*Cryopreservation and IVF protocols*	*Thawing and IVF steps*	*Volume added (μL)*	*Total vol. (μL)*	*Osmolality (mmol/kg)*
Draper–Moens Cryo	1. Thaw sample at 33°C	10		603
Draper–Moens IVF^11,12^ (2004–2008)	2. Add 70 μL HBSS	70		347
	3. Transfer sperm to eggs			
	4. Activate with 750 μL fish water	750		44
	5. Swirl to mix		830	
ZIRC 1				
Draper–Moens Cryo ZIRC IVF Version 1 (2009)	1. Thaw sample at 38°C	10		603
	2. Add 40 μL HBSS	40		367
	3. Transfer sperm to eggs			
	4. Activate with 320 μL dH_2_O	320		52
	5. No mixing		370	
ZIRC 2				
E400/RMMB Cryo ZIRC IVF Version 1 (2012)	1. Thaw sample at 38°C	20		547
	2. Add 40 μL HBSS	40		378
	3. Transfer sperm to eggs			
	4. Activate with 320 μL dH_2_O	320		54
	5. No mixing		380	
ZIRC 3 (current)				
E400/RMMB Cryo ZIRC IVF Version 2 (2014)	1. Thaw sample at 38°C	20		547
	2. Add 150 μL SS300	150		335
	3. Activate with 200 μL dH_2_O	200		142
	4. Transfer sperm to eggs			
	5. No mixing		370	

Key changes made to the sperm thawing and IVF procedures are detailed. Solution volumes and resulting osmolality at each step are shown. See also Figure 4.

HBSS, Hanks' balanced salt solution; IVF, *in vitro* fertilization; ZIRC, Zebrafish International Resource Center.

### IVF and test thaws

Females were isolated from males, and food was withheld the afternoon before egg collection. Egg collection and IVF were performed in the morning, within the first 2–3 h after the lights were turned on.^35^ To reduce anesthesia- and stripping-related mortality, females were sedated with 48 mg/L tricaine methanesulfonate (MS-222) for at least 10 min before anesthesia and egg collection.^36^ Before egg stripping, females were transferred to 168 mg/L tricaine methanesulfonate until fully anesthetized, rinsed in isotonic PBS, and gently dried on a paper towel. Eggs were collected into a 35 × 10 mm Petri dish.^10^ A sperm sample was removed from liquid nitrogen and thawed as described above. SS300 solution (150 μL at room temperature) was immediately added to the vial followed by 200 μL of dH_2_O to activate the sperm. The preactivated sperm was immediately transferred to the eggs by moving the pipette tip sideways into the eggs and expelling the sperm in a single motion. Eggs and sperm were left undisturbed for 2 min to ensure optimal fertilization. The dish was flooded with 0.5 × E2 embryo medium (7.5 mM NaCl, 0.25 mM KCl, 0.5 mM MgSO_4_, 0.5 mM CaCl_2_, 75 μM KH_2_PO_4_, 25 μM Na_2_HPO_4_, 0.35 mM NaHCO_3_, and 0.5 mg/L Methylene blue^35^). Fertilization rate was determined ∼3 h later, at the high stage of embryonic development.^37^ Fertilization rate was calculated as the number of developing embryos divided by the total number of eggs in the dish.

Before a fish line is considered cryopreserved at ZIRC, it must pass a quality-assessment test thaw. For test thaws, a representative sample of each line is used for IVF with AB wild-type eggs and for post-thaw motility analysis. A sperm sample is considered acceptable if at least 10% of eggs are fertilized. For motility assessment of test thaws, a small portion of the sperm solution was removed before activation. For the current ZIRC protocol (ZIRC 3, Table 2), 20 μL of the sperm/SS300 mixture was removed before activation and held in a microcentrifuge tube on ice for CASA. The volume of dH_2_O for activation was adjusted proportionately (to 176 μL) before transferring sperm into the eggs.

### Statistical analysis

**FIG. 1.**
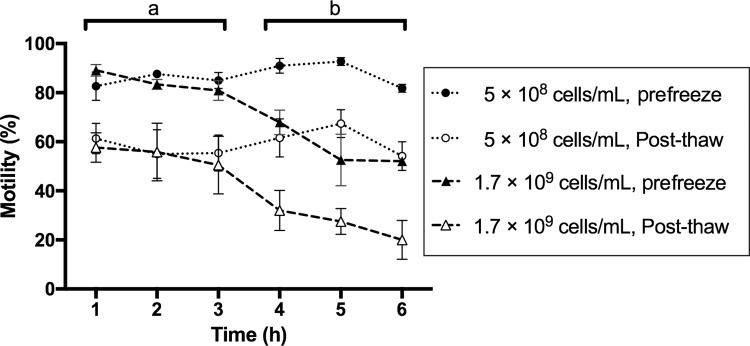
Sperm samples diluted in E400 can be held as long as 6 h on ice without significant loss of motility. Pooled sperm was collected and maintained in E400 extender as collected (1.7 × 10^9^ cells/mL) or further diluted 3.4-fold with E400 (to 5 × 10^8^ cells/mL) and held on ice for as long as 6 h. A portion of sperm from each group was cryopreserved hourly. Prefreeze and post-thaw sperm motility were analyzed for each time point. Percent motility of 1.7 × 10^9^ and 5 × 10^8^ cells/mL diluted sperm cells was plotted against storage time. The average percent motility of 1.7 × 10^9^ cells/mL sperm samples decreased over time (prefreeze, *black triangles*; post-thaw, *white triangles*). In contrast, sperm diluted to 5 × 10^8^ cells/mL with E400 (prefreeze, *black circles*; post-thaw, *white circles*) maintained motility for up to 6 h on ice. Data values labeled (**a**) were not significantly different for motilities of 5 × 10^8^ or 1.7 × 10^9^ cells/mL prefreeze (*black*) and post-thaw (*white*) samples during the first 3 h of storage on ice. However, all data values labeled (**b**) were significantly different between 5 × 10^8^ and 1.7 × 10^9^ cells/mL test groups between 4- and 6-h storage on ice, except for diluted post-thaw samples (*white circles*), which had comparable or greater motility than 1.7 × 10^9^ cells/mL prefreeze samples (*black triangles*). Two-way ANOVA *post hoc* statistics was calculated using Sidak's multiple comparison test. *p* < 0.001, *n* = 3 for prefreeze data; *n* = 9 for post-thaw data. ANOVA, analysis of variance.

**FIG. 2.**
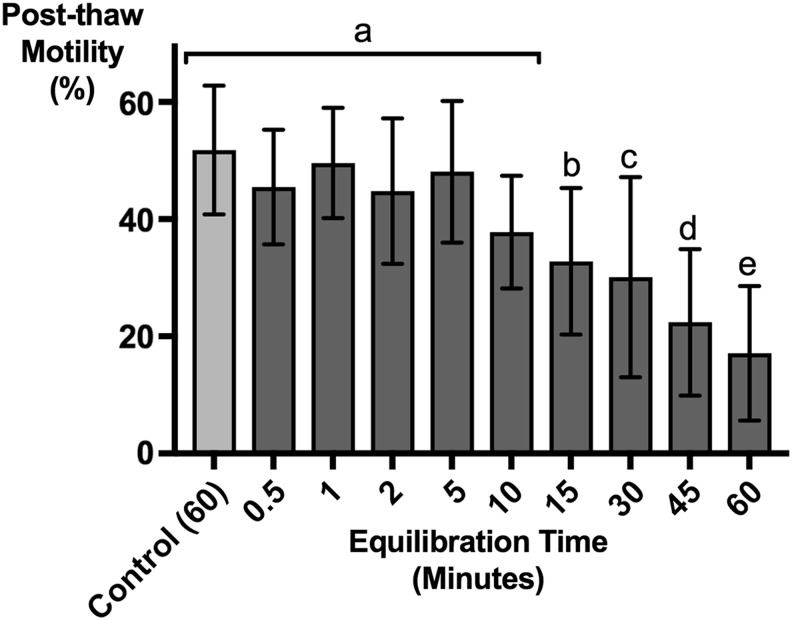
Sperm cells were held in RMMB CPM for at least 5 min without significant impact of solution toxicity on cell motility. Average post-thaw motility of sperm incubated with RMMB CPM at room temperature for various amounts of time before freezing is shown. The 60-min control was incubated for 60 min (in E400 without RMMB) at 25°C before adding RMMB and freezing with a 0.5-min equilibration time. Motility was assessd post-thawing, and an ordinary *post hoc* one-way ANOVA was performed using Tukey's multiple comparison test to the mean of each *column* with every other *column*. *n* = 27 for each of the test and control groups. No significant difference was found between control and 0.5–10 min test groups (labeled a). The average motility rate for the time points 15–60 min (labeled b, c, d, and e) was statistically significantly different to any of the average motilities labeled (a). The adjusted *p*-values for the control group versus 0.5, 1, 2, and 5 min test groups were 0.49<*p* < 0.9996, whereas *p* = 0.001 for control versus 10 min, and *p* < 0.0001 for control versus 15, 30, 45, and 60 min test groups. CPM, cryoprotective medium.

**FIG. 3.**
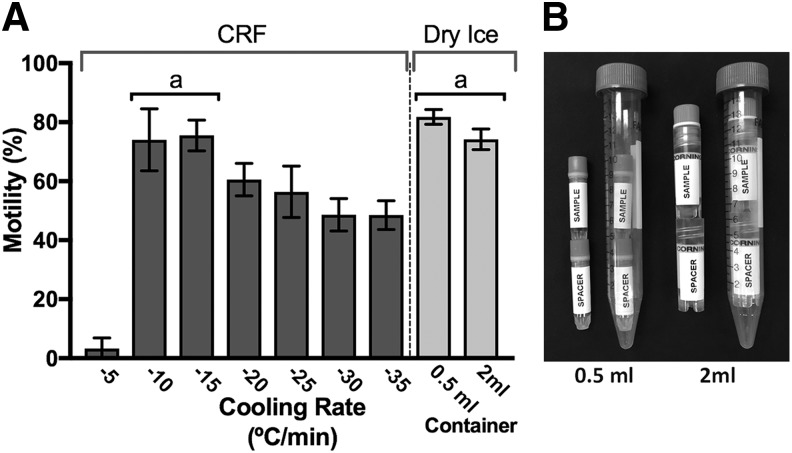
Controlled-rate cooling rates and resulting post-thaw motility can be reproduced with dry ice in two types of cryogenic vials. **(A)** *Dark gray bars*: post-thaw motility (mean ± SD) of sperm frozen in 0.5-mL matrix vials in a CRF. Cooling rates ranged from 5 to 35°C/min. An ordinary *post hoc* one-way ANOVA was performed using Tukey's multiple test to compare the mean of each *column* against every other *column*. The optimal cooling rate was identified as 10 to 15°C/min (labeled a). At these freeze rates, post-thaw motility was 74% ± 10% and 76% ± 5% (respectively, each *n* = 9), and these averages were not significantly different (*p* = 0.9998). However, all average post-thaw motilities of sperm frozen at rates of 5°C/min and between 20 and 35°C/min were significantly different (and lower). *Light gray bars*: post-thaw motility of sperm frozen in dry ice using 0.5-mL matrix vials (−14.1°C/min; 81% ± 2% motility, *n* = 9) and 2-mL Corning cryovials (−16.1°C/min; 74% ± 4% motility, *n* = 9). **(B)** Dry ice freezing vial assemblies. Cryogenic vial with sperm sample is placed in a 15-mL conical tube on *top* of an empty spacer vial of the same kind. (*Left*) 0.5-mL Matrix sample vial on *top* of an empty spacer Matrix vial *with* cap. (*Right*) 2-mL Corning cryogenic vials on *top* of an empty 2-mL spacer vial *without* cap. CRF, controlled-rate freezer; SD, standard deviation.

**FIG. 4.**
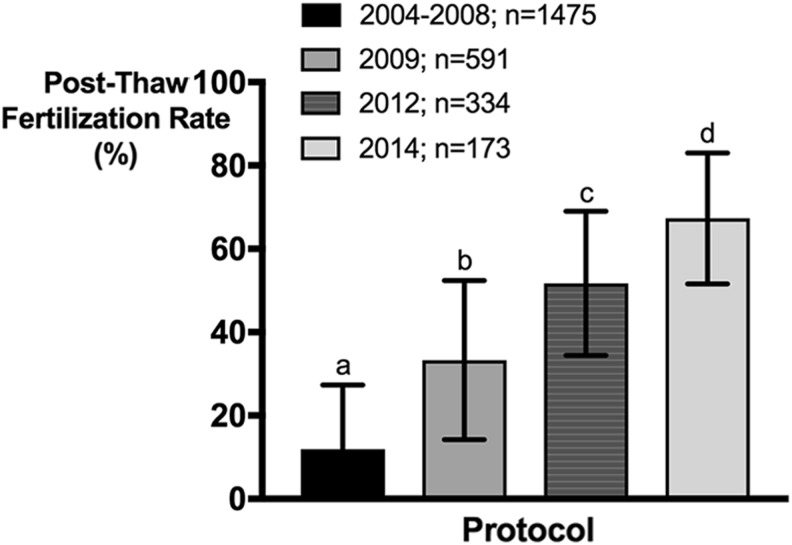
ZIRC test thaws suggest that successive implementation of protocol changes resulted in a stepwise increase in post-thaw fertilization rates. The cryopreservation and *in vitro* fertilization protocols utilized were: Draper and Moens (2004–2008, *n* = 1475); ZIRC 1 (2009, *n* = 591): Draper–Moens freeze protocol with reduced IVF volume and no mixing of sperm and eggs; ZIRC 2 (2012, *n* = 334): cryopreserved using E400, RMMB, and optimized freeze rate, same IVF protocol as ZIRC 1; and ZIRC 3 (2014, *n* = 173): same as ZIRC 2, but osmolality of fertilization solution was raised (Table 2). Post-thaw fertilization rates were obtained and averaged, and an ordinary *post hoc* one-way ANOVA was performed using Tukey's multiple test to compare the mean of each *column* against the average of every other *column*. 0.0001 > *p* > 0.00001; the averages of every *column* were significantly different from every other *column*. Cohen's *d* (size effect) was determined for all comparisons: a versus b, *d* = 1.29, b versus c, *d* = 1.0; c versus d, *d* = 0.93; a versus d, *d* = 3.59; a versus c, *d* = 2.53; a versus d, *d* = 3.59). IVF, *in vitro* fertilization; ZIRC, Zebrafish International Resource Center.

*Post hoc* statistical analysis was performed using Prism 7.0a software (GraphPad Software, Inc., La Jolla, CA). For sperm sample holding times in extender, we used two-way analysis of variance (ANOVA) with Sidak's multiple comparison test (Fig. 1). To evaluate sperm cell equilibration times in RMMB, optimal controlled-rate cooling rates, and average test-thaw fertilization rates, we used one-way ANOVA and Tukey's multiple comparison test (Figs. 2–4). Alpha was set to 0.001 in all cases. We also calculated whether sufficiently large sample sizes had been used (http://clincalc.com/stats/samplesize.aspx) and determined statistical power (http://clincalc.com/stats/Power.aspx) or size effect on results (using Microsoft Excel for Mac, v. 15.32) to ensure that results were significantly different from one another.

## Results

### Urine contamination of stripped sperm reduces osmolality and initiates motility

We tested the hypothesis that hypotonic urine prematurely activates zebrafish sperm during collection. Stripped sperm was collected from anesthetized males that had been thoroughly dried on a paper towel followed by drying of the urogenital opening with Kimwipe. Sperm cells were observed under a compound microscope without any added diluent. Despite all precautions to avoid exposing sperm to a low osmolality environment, we observed activated motile sperm cells in samples from 7 out of 10 males. By visual estimation, more than 50% of sperm cells were activated in five of these samples (*n* = 10). To test whether the premature activation resulted from the collected sample, we isolated the cell-free supernatant (seminal plasma) from stripped pooled sperm and found that the osmolality was below the activation threshold and in addition variable between replicates, measuring 214 mmol/kg (40 males) for the first and 159 mmol/kg (50 males) for the second replication (183 mmol/kg average). In contrast, we measured a blood plasma osmolality of 306 mmol/kg, which is consistent with previously reported zebrafish plasma osmolality of 296 ± 8^15^ and 315 ± 1 mmol/kg.^23^ These results suggest that urine contamination during collection of stripped sperm reduces osmolality, causes premature activation, and is a source of variability in sperm quality.

### Stability of pooled sperm in E400 extender

To test the efficacy of holding sperm in E400 extender before freezing, pooled sperm cells from 18 males were held at different concentrations in E400, and a portion of these suspensions was cryopreserved hourly for 6 h. Cell motility was tested before and after cryopreservation for each time point. When sperm was maintained on ice at 1.7 × 10^9^ cells/mL, a gradual decrease in prefreeze motility from 90% to 52%, and post-thaw motility from 58% to 20%, was observed (Fig. 1). However, when the sperm was further diluted 3.4 times to 5 × 10^8^ cells/mL with E400 and held on ice for as long as 6 h, we observed no significant decrease in prefreeze or post-thaw motility (Fig. 1). We obtained similar results when sperm from dissected testes was stored at different densities on ice for 6 h (data not shown).

### RMMB CPM equilibration time

To determine the sperm cell equilibration time in RMMB, sperm samples were incubated with RMMB for increasing amounts of time before freezing and then assessed for post-thaw motility with CASA. Post-thaw sperm motility was the same for sperm equilibrated for durations from 0.5 to 5 min with RMMB (Fig. 2; *n* = 9). Although average motility was lower after 10 min of equilibration, the difference was not statistically significant, suggesting that sperm can be equilibrated for as long as 10 min in RMMB without significant reduction of post-thaw motility. Incubation times greater than 10 min, however, resulted in a gradual decrease of post-thaw motility. In contrast, the post-thaw motility of the 60-min control sperm, which was maintained at room temperature for 60 min in E400 without RMMB and equilibrated for 0.5 min with RMMB just before freezing, was unaffected (Fig. 2, control).

### Determination of the optimal cooling rate

To determine the optimal cooling rate for RMMB and 0.5-mL Matrix and 2-mL Corning cryogenic vials, samples were cooled in a CRF at programmed rates from 5 to 35°C/min. Motility of the pooled sperm before freezing was 96%–98% for all rates (Table 1). Cooling at 15°C/min resulted in a post-thaw motility of 75% ± 5% (*n* = 3) and a motile cell survival of 28% (Table 1 and Fig. 3A). Cooling at 10°C/min resulted in 74% ± 10% post-thaw motility (*n* = 3) and motile cell survival of 31%. Because most laboratories do not have access to a CRF, the cooling rate was reproduced with dry ice for 0.5-mL Matrix and 2-mL Corning cryogenic vials held in the dry ice within a 15-mL conical centrifuge tube containing a spacer vial in the bottom (Fig. 3B). Measured cooling rates were 14.1°C/min for the 0.5-mL Matrix vial and 16.1°C/min for the 2-mL Corning vial. Thawed samples from Matrix vials frozen in dry ice had a post-thaw motility of 82% ± 2% (*n* = 3) and the highest motile-cell survival (45%). Samples frozen in the Corning cryogenic vials had a post-thaw motility of 74% ± 4% (*n* = 3) and 39% motile cell survival.

### Mixing of eggs and sperm after activation reduces the fertilization rate

We found that mixing sperm and eggs after activation by swirling the Petri dish during IVF resulted in a significantly lower percentage of fertilized eggs compared to undisturbed dishes. When eggs and sperm were swirled for 10 s after activation, fertilization rates averaged at 14.3% ± 14.1% (*n* = 20). In contrast, when eggs and sperm were left undisturbed for 2 min, fertilization rates were 39.6% ± 15.3% (*n* = 20).

### Stepwise improvement of post-thaw fertilization with implementation of protocol changes

Stepwise implementation of E400 and RMMB and the modifications to the cryopreservation, thawing, and IVF processes have resulted in progressive improvements of post-thaw fertilization rates in cryopreserved lines (Fig. 4). Table 2 summarizes key protocol changes, solution volumes, and resulting osmolality at each step from thawing to sperm activation. The original Draper–Moens freezing and IVF protocols^11,12^ used at ZIRC yielded an average fertilization rate of 12% ± 15% (Fig. 4), and 45% of test thaws failed (<5% fertilization). Initial changes made to the thawing and IVF protocol in 2009 (ZIRC 1, Table 2) increased the average fertilization rate to 33% ± 19% (Fig. 4). These changes included increasing the thawing rate by increasing the water bath temperature, increasing the effective concentration of sperm around the eggs by reducing the overall fertilization volume, and eliminating the swirling of the Petri dish during the fertilization period. Switching to the E400/RMMB cryopreservation protocol (in 2012, ZIRC 2, Table 2) resulted in further improvement of the average fertilization rate to 52% ± 17%. As a final step, we increased the osmolality at sperm activation (in 2014; ZIRC 3, Table 2). The current E400/RMMB cryopreservation and IVF protocol (ZIRC 3) resulted in an average fertilization rate of 67% ± 16%.

In 2016, 121 lines were frozen at ZIRC with the current protocol (ZIRC 3, Table 2). Prefreeze and post-thaw CASA data and IVF results of test thaw QAs are summarized in Table 3. The average post-thaw motility was 20% ± 13%; the average post-thaw motile sperm count was 3.1 ± 3.4 × 10^6^ cells/sample and resulted in an average fertilization rate of 68% ± 16%.

**Table 3.** Computer-Assisted Sperm Analysis Has Been Implemented as a Quality Assessment Tool for Routine Cryopreservation at ZIRC

*Sperm samples (*n* = 121)*	*% Motile*	*Motile cell conc. (M/mL)*	*Motile cell count (M/sample)*	*Motile, mean VAP*	*Motile, mean VSL*	*Total cell conc. (M/mL)*	*Total cell count (M/sample)*	*IVF avg. fertility (%)*
Prefreeze	71.7 ± 19.2	1098.6 ± 826.7	22.0 ± 16.5	99.4 ± 12.0	93.9 ± 11.4	1403.1 ± 859.3	28.1 ± 17.2	—
Post-thaw	20.1 ± 12.9	153.4 ± 169.6	3.1 ± 3.4	62.4 ± 13.2	59.4 ± 13.1	679.4 ± 470.4	13.6 ± 9.4	68.4 ± 15.6

QA of cryopreserved fish lines using the new E400/RMMB cryopreservation protocol, pooled sperm, and freezing in dry ice. CASA data of prefreeze and thawed sperm (*n* = 121, November 2015 to September 2016) and fertilization rates (at 3 hpf) of test thaws are shown (mean ± SD).

CASA, computer-assisted sperm analysis; M, million; QA, quality assessment; SD, standard deviation; VAP, average path velocity; VSL, straight-line velocity.

We developed a protocol based on our results that includes all changes to the cryopreservation method and offers experimental flexibility for multiple applications. We will continue to refine details, and the most recent updates to the protocol can be downloaded from the ZIRC site here: http://zebrafish.org/documents/protocols/pdf/Cryopreservation_IVF/zirc_rmmb_freezing_protocol.pdf

## Discussion

We conducted a stepwise and iterative evaluation of the cryopreservation process, from sperm collection to post-thaw IVF, introducing modifications that resulted in higher post-thaw motility and fertilization rates. Specifically, we: (1) increased the osmolality of extender used to suspend sperm samples after collection, (2) developed a new extender (E400) and CPM (RMMB), (3) introduced QA for cell density and motility, (4) determined the optimal cooling rate, and (5) implemented a new thawing and IVF procedure. Overall, these protocol changes significantly improved post-thaw fertilization rates (Fig. 4).

QA of processes and quality control (QC) of materials can be applied at many levels of the cryopreservation pathway, starting with the conditioning of males in preparation for sperm collection through the cryopreservation method, to the eggs used for IVF after thawing of sperm samples.^27,38^ QA and QC activities are the foundation for efficient preservation and recovery of cryopreserved materials and for the reproducibility of results. In this study, we focused on sperm cell density and motility assessment to develop a cryopreservation protocol with higher post-thaw fertilization rates. Cell density measurements and regulation of cell density during cryopreservation will be particularly useful to disseminate a reproducible and reliable protocol to other laboratories and develop community-wide standards. Previous zebrafish sperm cryopreservation methods did not include cell-density or motility measurements for QC.^9,11,12,14^ The original protocol^9^ was developed at a time when the necessary equipment either did not exist or was costly to procure. The only options were to count cells with a hemocytometer and observe motility under a microscope. Although these methods are useful for establishing reference densities and to calibrate equipment such as spectrophotometers, they are relatively time consuming and, thus, impractical for routine use.

Another hindrance to QA was that samples were frozen from individual males, resulting in small collection volumes (0.5–2 μL). An initial dilution (i.e., normalizing) did not produce enough sample volume for analysis. In this study, we implemented QA measures, for which pooling sperm was particularly valuable, allowing for NanoDrop optical density measurements and CASA to quantify cell density and sperm motility. Although these measures require additional steps, they have been integrated into the work flow and do not significantly affect sample throughput. After collection in E400, sperm can be held on ice for 3–6 h depending on dilution (Fig. 1), providing sufficient time for quality analysis. When samples are pooled from multiple males, sperm volumes are greater, and cell density results are representative for the entire pool of frozen samples. The extra effort spent on measuring cell density and adjusting cell concentrations mitigates male-to-male variability and leads to a more optimized use of collected sperm and better reproducibility of post-thaw results. Aspects of fish husbandry such as animal age, health status, holding density, and feeding are also expected to affect animal and sperm cell quality,^7,27,38–40^ but are not reported in this study.

Freshwater fish produce copious amounts of dilute urine.^41^ Both the distal mesonephric duct and the spermatic duct terminate at the urogenital sinus and release urine and sperm, respectively, through the urogenital pore.^22,25,42,43^ The spermatozoa of freshwater fishes are immotile in testes and seminal plasma and become activated at spawning when released into the aqueous environment.^17,18,44^ Seminal plasma has an osmolality similar to blood plasma,^32,45^ and zebrafish sperm cells are immotile at isotonic and hypertonic osmolalities. Zebrafish sperm has been shown to be activated at or below 270–288 mmol/kg.^15,23^ We found that the osmolality of cell-free supernatant (seminal plasma) from zebrafish sperm collected by stripping was between 159 and 214 mmol/kg, well below the osmolality of blood plasma (306 mmol/kg). This indicated that the stripped sperm was contaminated with hypotonic urine and, therefore, prematurely activated during the stripping process.

Because the motility of zebrafish sperm can be started, stopped, and restarted by adjusting the extender osmolality,^20–22^ we designed a new extender, E400, a buffered, hypertonic saline solution (400 mmol/kg). Hypo-osmotic contamination of sperm collected by stripping activated the sperm in 7 out of 10 samples. Due to its high osmolality, the E400 extender was able to counteract urine contamination immediately after collection and immobilize sperm cells and maintain sperm in an immobilized state. Using E400 allows for pooling of sperm from multiple males and provides ample time for QA before cryopreservation.

Potassium is a key component of cyprinid seminal plasma,^20,32,46^ and incubation of sperm in a medium that contains potassium has been shown to increase and regenerate sperm motility.^21,22,32,47^ Because of its beneficial characteristics, we tested and increased the amount of potassium in E400 (130 mM) compared to previous extender solutions for zebrafish sperm.^9,12,14,15^ Glucose was also tested and added to E400 because it has been identified as a component of seminal plasma in carp and other freshwater cyprinids.^46,48,49^ Exposure of zebrafish sperm to exogenous organics, including glucose, in an activating solution was shown to have no metabolic role in motile sperm. However, the incubation of quiescent (immotile) sperm with an adenosine triphosphate synthesis inhibitor (2,4-dinitrophenol) before activation resulted in a marked decrease (67%) in motile sperm postactivation.^50^ A progressive decrease of ATP content was observed in immobilized carp spermatozoa exposed to an inhibitor of mitochondrial respiration (10 mM KCN).^51^ It is currently unknown whether glucose is used as an energy source in quiescent zebrafish sperm, but these results suggest that ATP production in quiescent sperm is necessary for adequate motility upon activation.

Overall, we developed the E400 from previously published extenders, to include glucose^15^ and to be similar to Carp seminal plasma.^14^ We further increased the potassium to raise the osmolality for sperm activation control and used HEPES buffer for better pH control at ultralow temperatures.^33^

Our results suggest that zebrafish sperm survives in E400 on ice for several hours. Normalized and pooled sperm (at 1.7 × 10^9^ cells/mL) could be maintained on ice for 3 h in E400, whereas sperm diluted 3.4 × with E400 to 5 × 10^8^ cells/mL was held for as long as 6 h without significant reduction of prefreeze or post-thaw motility. These observations indicate that cell densities in an extender need to be adjusted carefully to match the limited availability of the solution components that equilibrate across cell membranes. By fine-tuning cell densities and the cryoprotective features of the extender, ultimately, post-thaw survival and fertilization rates can be optimized, with the added benefit of maximizing the number of samples generated per male.

The CPM RMMB contains a zwitterionic buffer, bicine (pH 8.0), that is stable at low temperatures.^33,52^ Skim milk powder (2.5%) is included in RMMB to decrease the tail-to-tail agglutination common with zebrafish sperm and is thought to have a protective effect during cryopreservation. Milk is a complex physiologic medium and its mechanism of action during cryopreservation is not well understood. However, several studies indicate that casein micelles are the active components in milk that protect sperm by preventing cholesterol and lipid loss from sperm cell membranes.^53–56^ The methanol concentration in the RMMB medium was reduced (5% v/v sample conc.) compared to previous methods^9,15^ to minimize cell toxicity. We found that the post-thaw motility of sperm that had been equilibrated for more than 10 min with RMMB was reduced (Fig. 2), indicating some toxicity to sperm cells. The RMMB medium has a relatively high osmolality, averaging 575 mmol/kg. High osmolality has been shown to dehydrate cells before freezing.^57^ The main contribution to the hypertonicity of the medium stems from the 20% (w/v) raffinose. To minimize osmotic damage during the addition of the RMMB,^16,58^ sperm should be mixed with RMMB just before freezing, and samples should be aliquoted and ready to freeze within 10 min.

In different cryoprotectants, sperm can have different optimal cooling rates, above or below which cells are damaged by intra- and extracellular ice crystal formation.^16,58^ To determine the optimal cooling rate for the E400/RMMB protocol, we froze samples in cryogenic vials at cooling rates ranging from 5 to 35°C/min in a CRF. Based on resulting post-thaw sperm motility, we found that the optimal cooling rate was between 10 and 15°C/min (Table 1 and Fig. 3A).

For most laboratories, freezing samples in dry ice is advantageous because it is readily available and offers flexibility in work flow and the timing of sample freezing compared to a CRF. Larger quantities can be processed at once in a CRF. Samples can be added to dry ice at any time and removed after 20 to 60 min. This is particularly advantageous when freezing samples from single males. We reproduced the CRF cooling rate of 15°C/min in dry ice by adding the sample-containing cryogenic vial to a 15-mL conical centrifuge tube containing an additional empty cryogenic vial as a spacer (Fig. 3B). Depending on the type of cryogenic vial, this resulted in cooling rates of 14 to 16°C/min and post-thaw motility above 70% (Table 1 and Fig. 3A).

Because the thawing and activation of sperm can introduce cryogenic and osmotic damage to cells,^16,58,59^ we also optimized thawing and IVF protocols to improve post-thaw fertilization. Two new solutions were introduced. Previously, Hanks' balanced salt solution (HBSS) was utilized to dilute the CPM immediately after thawing sperm samples. HBSS solutions commonly found in zebrafish laboratories have variable recipes. The osmolality is typically less than 300 mmol/kg, and the bicarbonate in HBSS has a low buffering capacity and is unstable over time because the release of CO_2_ causes the pH to rise.^60^ HBSS was replaced with a buffered, physiologic saline solution, SS300, that was designed to have an osmolality similar to zebrafish blood plasma (≈300 mmol/kg). In addition, an isotonic PBS rinse was introduced for use with females, after removal from the anesthetic solution and before egg collection (can also be used for males before stripping). Because fish cannot be dried completely, the isotonic rinse was incorporated to prevent the unintended activation of eggs (or sperm) during collection. Like sperm, zebrafish eggs are activated by the spawning medium.^61^ With or without the presence of sperm, activated eggs undergo a programmed series of surface rearrangements, including elevation of the chorion and cortical-granule exocytosis. Within 60 s of egg activation, a plug of material from the cortical-granule reaction develops and blocks the micropyle.^62–64^

The initial changes made to the thawing and IVF protocol included increasing the thawing rate, reducing the overall solution volume, and elimination of mixing during fertilization (ZIRC 1, Table 2). Previous IVF protocols specified swirling the Petri dish to mix eggs and sperm immediately after activation.^12,13^ With these changes, an approximate 20% improvement was seen in fertilization rates (Fig. 4). When these protocol changes were evaluated individually, our observations indicated that the improvement in fertilization (39.6% vs. 14.3%) was primarily due to the absence of swirling after activation. We speculate that swirling the sperm-egg mixture causes a disruption in the ovarian fluid surrounding the eggs. Ovarian fluid has been shown to prolong sperm motility and enhance fertilization.^65–70^ The ovarian fluid contribution to solution osmolality in the immediate vicinity of the eggs may have pronounced effects on motility of the more fragile cryopreserved sperm cells.

We further modified the thawing and IVF protocol to reduce the osmotic stress experienced by the sperm cells. The starting osmolality of a thawed sample is ∼550 mmol/kg. The volume of solutions added after thawing (SS300 and dH_2_O) was adjusted to raise the final osmolality at sperm activation by ∼90 mmol/kg (to ∼140 mmol/kg; ZIRC 3, Table 2), thus providing less of an osmotic shock. Table 2 summarizes the changes in osmolality following each solution addition. The higher osmolality at sperm activation with the current (ZIRC 3) protocol results in a momentary delay in initiation of sperm motility.^65^ Activating the sperm before it is transferred to the eggs ensures that the sperm are motile at the time of egg activation. The total solution volume added to eggs (370 μL) is sufficient to cover several clutches of pooled eggs for 2 min, after which fertilization is complete, and the eggs are flooded with embryo medium.

This study offers new methods for zebrafish sperm cryopreservation and IVF that are easily adaptable for a wide range of research and facility applications. The E400/RMMB protocol was developed and optimized for freezing sperm from zebrafish with an AB wild-type background. Zebrafish with different genetic backgrounds may require further protocol modifications to obtain similar post-thaw fertilization rates, and ZIRC will continue to study this. Updates to the protocol will be posted in the protocol section of the ZIRC web site (http://zebrafish.org/documents/protocols.php).
